# Supplementary material for: Test of the Rehabilitation Goal Screening (ReGoS) Tool to Support Decision Making and Goal Setting in Physical and Rehabilitation Medicine Practice
Source: Int J Environ Res Public Health. 2022 Nov 23;19(23):15562. doi: 10.3390/ijerph192315562 (PMC9738768; doi:10.3390/ijerph192315562)
Supplement: Supplementary file 1 [file ijerph-19-15562-s001.zip › ijerph-1970654-supplementary.pdf]

International Journal of
Environmental Research and Public Health

MDPI

Supplementary Materials

Test of the Rehabilitation Goal Screening (ReGoS) Tool to Support Decision Making and Goal Setting in Physical and Rehabilitation Medicine Practice

Hannover Medical School
Department of Rehabilitation Medicine

Rehabilitation Goal Screening (ReGoS)

Patient code: sex: female O male O		time t1 O t2 O t3 O										
Date of birth: employed: yes O no O		Date:										
		**0 = no problem / no limitation / no disability / no pain<br>10 = maximal problem / maximal limitation / maximal disability / maximal pain**										
**No**	**Question**	**0**	**1**	**2**	**3**	**4**	**5**	**6**	**7**	**8**	**9**	**10**
1	Do you have any limitations of your energy and drive because of your actual disease? Please rate the intensity of these limitations.	O	O	O	O	O	O	O	O	O	O	O
2	Do you have sleep disturbances because of your disease? Please rate the intensity of this problem.	O	O	O	O	O	O	O	O	O	O	O
3	Do you feel emotionally disturbed because of your disease? Please rate the intensity of these disturbances.	O	O	O	O	O	O	O	O	O	O	O
4	How strong is your pain?	O	O	O	O	O	O	O	O	O	O	O
5	Are you limited in doing your daily activities due to your disease? Please rate the intensity of this problem.	O	O	O	O	O	O	O	O	O	O	O
6	Do you feel limitations in handling stress and psychological demands because of your actual disaease?	O	O	O	O	O	O	O	O	O	O	O
7	Do you have problems with changing your body position because of your disease (e.g. getting out of bed or sitting down on a chair)? Please rate the intensity of this problem.	O	O	O	O	O	O	O	O	O	O	O
8	Do you have any problems with walking due to your disease? Please rate the intensity of this problem.	O	O	O	O	O	O	O	O	O	O	O
9	Do you have problems using public transportation because of your disease? Please rate the intensity of this problem.	O	O	O	O	O	O	O	O	O	O	O
10	Do you have problems washing yourself because of your disease? Please rate the intensity of this problem.	O	O	O	O	O	O	O	O	O	O	O
11	Do you have problems using the toilet because of your disease? Please rate the intensity of this problem.	O	O	O	O	O	O	O	O	O	O	O
12	Do you have problems dressing and undressing yourself because of your disease? Please rate the intensity of this problem.	O	O	O	O	O	O	O	O	O	O	O

Hannover Medical School
Department of Rehabilitation Medicine

Rehabilitation Goal Screening (ReGoS)

13	Do you have problems eating by yourself because of your disease (e.g. holding a fork, cutting with a knife)? Please rate the intensity of this problem.	o	o	o	o	o	o	o	o	o	o	o
14	Do you have problems managing your household because of your disease? Please rate the intensity of these problems.	o	o	o	o	o	o	o	o	o	o	o
15	Do you have problems participating in work because of your disease? Please rate the intensity of this problem.	o	o	o	o	o	o	o	o	o	o	o
16	Are you limited in doing leisure and recreational activities due to your disease? Please rate the intensity of this problem.	o	o	o	o	o	o	o	o	o	o	o
17	Which function or activity has the greatest limitation because of your disease? Please describe with a few key words.											
18	Please rate how severely this limitation is.	o	o	o	o	o	o	o	o	o	o	o
19	Which function or activity is your most important goal of treatment? Please describe with some key words.											
20	Please rate how severely this function or activity is restricted.	o	o	o	o	o	o	o	o	o	o	o

Figure S1. Rehabilitation Goal Screening (ReGoS) Tool.
